# The *FTO* variant conferring enhanced UCP1 expression is linked to human migration out of Africa

**DOI:** 10.1093/lifemeta/loae027

**Published:** 2024-06-22

**Authors:** Nan Yin, Dan Zhang, Jiqiu Wang

**Affiliations:** Department of Endocrine and Metabolic Diseases, Shanghai Institute of Endocrine and Metabolic Diseases, Ruijin Hospital, Shanghai Jiao Tong University School of Medicine, Shanghai 200025, China; Shanghai National Clinical Research Center for Metabolic Diseases, Key Laboratory for Endocrine and Metabolic Diseases of the National Health Commission of the People’s Republic of China, Shanghai National Center for Translational Medicine, Shanghai 200025, China; Department of Pediatric Endocrine and Metabolic Diseases, Shengjing Hospital of China Medical University, Shenyang, Liaoning 110801, China; Department of Endocrine and Metabolic Diseases, Shanghai Institute of Endocrine and Metabolic Diseases, Ruijin Hospital, Shanghai Jiao Tong University School of Medicine, Shanghai 200025, China; Shanghai National Clinical Research Center for Metabolic Diseases, Key Laboratory for Endocrine and Metabolic Diseases of the National Health Commission of the People’s Republic of China, Shanghai National Center for Translational Medicine, Shanghai 200025, China

**Dear Editor,**

Genome-wide association studies (GWAS) revealed a large number of common variants in the human genome associated with an increased risk of diseases. The lead signals of common obesity-associated variants are located within the first intron of the *FTO* (fat mass and obesity-associated) gene, being the most significant and impactive single-nucleotide polymorphism (SNP) clusters in obesity inheritance [1]. Although a strong positive association between these *FTO* “risk SNPs” (including rs1421085 T>C) and obesity is reported across human populations of diverse ancestry, relatively weak (even none) significant associations have been observed in African populations [2]. As known, the frequency of the *FTO* SNPs varies among different ancestral groups, with the “risk allele” being prevalent in individuals of European ancestry and less common in Asian and African populations. Notably, the linkage disequilibrium (LD) of the “risk SNPs” in African populations was much lower than in non-African populations [3]. The reason for the discrepancy remains elusive thus far; however, it could be redefined in the context of the unexpected biological function of the *FTO* SNPs *in vivo* [4].

Very recently, by constructing global and brown adipocyte-specific knock-in mouse models harboring the homologous human rs1421085 T>C variant, we found that mice carrying the homozygous CC-alleles exhibit enhanced brown adipose tissue (BAT) thermogenesis and resistance to high-fat diet-induced obesity under room temperature (22°C, active BAT), while these obesity-related metabolic phenotypes were compromised under thermoneutral (30°C, silent BAT) condition [4]. More importantly, mice harboring the CC-alleles showed approximately 6°C higher than those with TT-alleles in a cold room (4°C for 4 h). Thus, the rs1421085 T>C variant was capable of enhancing thermogenesis and promoting resistance to cold in the presence of active BAT [4]. Considering the biological roles and geographical distribution patterns of this variant, ambient environmental temperature appears to be a pivotal factor influencing the frequency of the variant across the planet.

Climatic shifts exert evolutionary forces that are instrumental in molding phenotypic diversity across and within different species. Under selective pressure, genes responsible for phenotypes with survival advantages may carry detectable signatures of selection in the spatial patterns of genetic variation. For instance, the *EDAR* p.V370A mutation is selected by a warm and humid Asian environment [5], and the unique *EPAS1* (endothelial Per-Arnt-Sim domain protein 1) haplotype structure is selected by the hypoxic environment of the high-altitude Tibetan plateau [6]. Previous studies have reported that enhanced thermogenesis provides survival advantages during periods of nocturnal or hibernal cold and the cold stress of birth [7]. Genomic variations conferring enhanced cold resistance may experience preferential selection in cold climates. Building upon these existing foundations, we aimed to investigate whether the frequency of rs1421085_C-allele is related to ambient temperature. We proposed a hypothesis that the differential distribution of the C-allele among populations from Africa to Eurasia may be dependent on positive selection by cold stress pressure.

*UCP1* (uncoupling protein 1) is one of the most critical genes involved in thermogenesis and resistance to environmental coldness. As we recently revealed that the homologous rs1421085 T>C variant increases the expression of *UCP1* in rodent BAT, we here collected a number of human fetal BAT samples to explore the potential changes of UCP1 in the rs1421085_C genotype compared with the rs1421085_T genotype in humans. A significantly enhanced UCP1 immunofluorescence staining was detected in the BAT of TC-allele carriers when compared with TT-allele carriers (Fig. 1a and b). With an enlarged sample size (*n* = 45), a robust increase (~3 folds) of *UCP1* mRNA expression was observed in the TC-alleles (Fig. 1c). These human data are highly consistent with our recent findings in mice [4].

**Figure 1:**
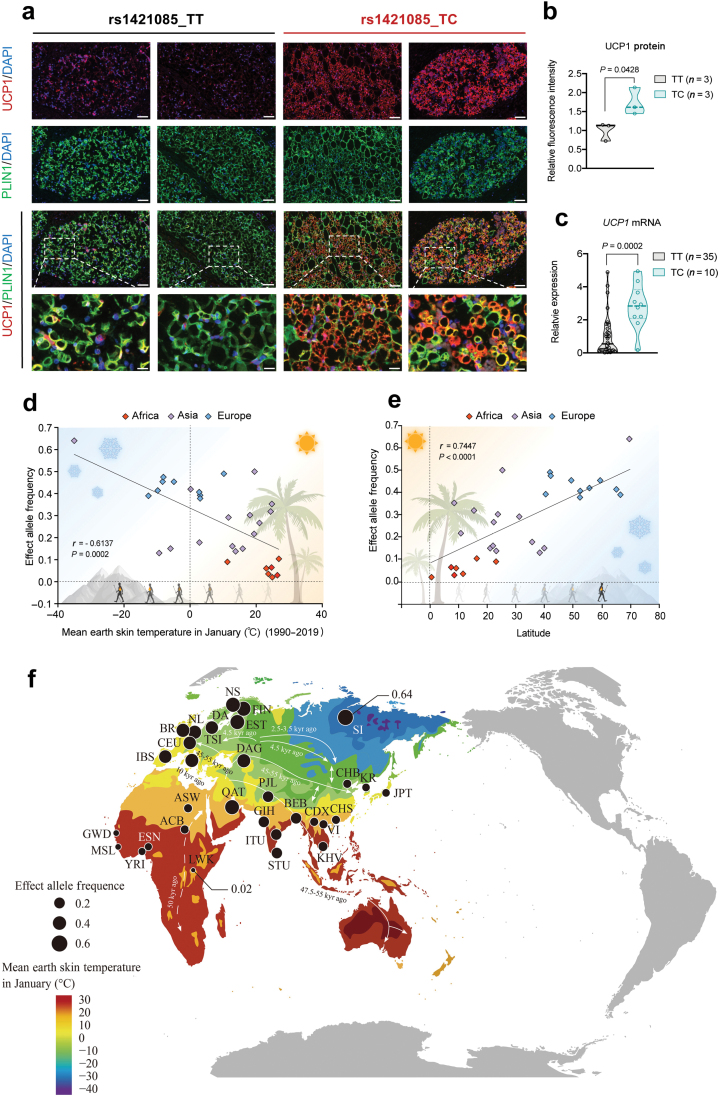
The *FTO* rs1421085 T>C variant enhances UCP1 expression and traces human migration out of Africa. (a) The representative immunofluorescence images of UCP1 (red) in human fetal brown fat, in which the nuclei are stained with 4ʹ-6-diamidino-2-phenylindole (DAPI) (blue) and lipid droplets with Perilipin 1 (PLIN1) (green) (scale bar = 100 μm for the top 3 lines, and scale bar = 25 μm for the bottom enlarged panels). (b) Quantification analysis of UCP1 protein. For each BAT sample, four visual fields were randomly selected for fluorescence intensity quantification, and the average values were used for statistical analysis. A total of 3:3 samples were included in the analysis. (c) The mRNA expression level of *UCP1* in the two genotypes. A total of 45 samples (35 in the TT-alleles and 10 in the TC-alleles) were included in the analysis. (d and e) Correlation analysis of the frequency of the rs1421085_C variant and mean earth skin temperature in January (d) or absolute latitude (e). The icons of panel d and e were provided by BioRender. (f) The migration route of modern humans together with a genetic diffusion pattern of the rs1421085 T>C variant on the African and the Eurasian continents. (The work of Nielsen *et al*. [9] was adapted to label the migration route time). Each circle represents a distinct population, circle size indicates the frequency of rs1421085_C allele, and map color represents the mean earth skin temperature in January. Arrows indicate the directions of population migration.

We next analyzed the association between the allele frequency of the rs1421085 T>C variant in various populations and ambient temperatures across Eurasian-African continents, and we observed a significant inverse correlation between the C-allele frequency and mean earth skin temperatures in January among 31 distinct populations (*P = *0.0002, *r* = −0.6187) (Fig. 1d). The inverse correlation remained significant even after the adjustment for Paleozoic temperature, which might once affect BAT function of ancient humans, by using two established models [8] (*P* < 0.0001, *r* = −0.6620, and *P* < 0.0001, *r* = −0.6561, respectively) (Supplementary Fig. S1a and b). After modern *Homo sapiens* migrated out of Africa and eventually settled on the Eurasian continent, their ambient temperature/residential location shifted from a warm/low latitude to a cold/high latitude. We further analyzed the correlation between the variant frequency and the latitude coordinates, a determinant factor of ambient environment temperature, across different populations and observed a significant positive association (*P* < 0.0001, *r* = 0.7447) (Fig. 1e). In contrast, no statistically significant correlation was found between the variant frequency and longitudes or altitudes among populations (Supplementary Fig. S1c and d). Selective sweep often results in pronounced patterns of LD near loci that offer adaptive advantages to environmental stresses. Furthermore, we performed LD structure analysis on all African, Asian, and European ancestry populations in the 1000 Genomes Project (1KGP) and found that the number of SNPs highly linked to rs1421085 (*R*² > 0.8) was the highest in European populations, followed by Asian populations, and the lowest in African populations (Supplementary Fig. S2a). SNPs surrounding the rs1421085 locus present a more significant pattern of LD in individuals of European descent (represented by Utah residents with Northern and Western European ancestry, CEU) than in those of African descent (represented by Yoruba in Ibadan, YRI) from equatorial regions, with the patterns observed in individuals of Asian descent (represented by Han Chinese in Beijing, CHB) falling intermediate to the two populations (Supplementary Fig. S2 b–d). Referring to modern human migration route maps reported by a previous study [9], we devised a possible genetic dispersion model for the rs1421085 T>C variant from the African to Eurasian continents. According to this model, the C-allele frequency exhibited a temperature-dependent (but not random) distribution pattern across these continents, which was found to be the lowest in East Africa (~2%), intermediate in East Asia, higher in Europe, and reached its peak in Siberia (~64%) (Fig. 1f; Supplementary Table S1).

Of note, this correlation should not be interpreted as a definitive causal linkage between the two variables (allele frequency and temperature), which requires further support from archaeological or genetic evidence of ancient *Homo sapiens*. Several possibilities warrant consideration, which include direct selection, indirect selection, and voluntary selection.

Direct selection means that C-allele carriers yielded more powerful survival and/or reproductive advantages in cold environments, resulting in a relative enrichment of this variation in cold areas. From the perspective of direct selection, it is important to delve into the origin of *Homo sapiens* and the evolutionary dynamics that transpired. Genetic analyses of modern *Homo sapiens* reveal that Africans exhibit the highest genetic diversity, with populations from other continents forming subsets of the African gene pool. Consequently, it is inferred that modern *Homo sapiens* originated from Africa and subsequently migrated to diverse regions, known as the Out-of-Africa (OOA) hypothesis [10]. As predicted, the rs1421085 T>C mutation may first appear in the human genome approximately 9140 generations ago (95% confidential interval, 7,771–10,961 generations), that is, about 265,000 years ago (29 years/generation) [11]. The absence of the rs1421085_C-allele in all currently available Neanderthal and Denisovan genomes further substantiates the recent evolutionary emergence of this variant. The occurrence of this variant aligns with the late Middle Pleistocene spanning from around 780,000 to 130,000 years ago, a period characterized by the repeated widespread formation of ice sheets and glaciers in regions of high latitude and mountainous topography. These dramatic climatic oscillations exerted substantial ecological and evolutionary impacts, posing huge challenges to the migration of early *Homo sapiens* out of Africa. In this context, environmental selection serves as a pivotal factor in shaping evolutionary processes, and genomic variations that confer enhanced cold resistance may undergo preferential selection. The functional *FTO* variant—rs1421085 T>C—may offer the newborn carriers a significant survival advantage in cold environments, particularly during the short term after birth, by enhancing BAT thermogenesis.

We speculated that this mutation likely emerged as isolated or occasional events when small bands of ancient *Homo sapiens* ventured out of Africa and encountered the harsh cold. The frequency of the mutation then potentially increased as the population expanded gradually within specific localized areas. However, the planet has experienced multiple glacial-interglacial alternations frequently, and *Homo sapiens* who have migrated outwards have been forced to retreat to warm Africa several times due to extreme cold [12]. In this context, the mutation in the *FTO* gene may have reintegrated into the African gene pool during the out-and-back process. With the onset of the subsequent interglacial phase and Earth’s warming, *Homo sapiens* once again embarked on a new exodus, leaving Africa to migrate and colonize other continents. After numerous episodes of migration and retreat, modern *Homo sapiens* ultimately spread across multiple continents [13], thereby ensuring the preservation and persistence of this variant within the genomes of continental populations. The C-allele has undergone long-standing and variable levels of positive selection, a process shaped by disparate environmental temperatures among the continents. This selective pressure is likely to culminate in a heterogeneous distribution of the C-allele across currently various populations. Although the C-allele frequency overall correlates strongly with temperature, there are some deviations observed in West and South Asia. One possibility is that multiple migrations of populations with Caucasian ancestry reversed the C-allele frequency in these regions by introducing European genetic heritage, which typically exhibits a higher frequency of the C-allele [14, 15]. However, the complicated underlying reasons warrant further exploration.

Moreover, enhanced brown fat thermogenesis may not be the exclusive factor enhancing human survival in cold environments. In adults, thermal homeostasis is principally maintained by increasing the thickness of the insulation layer (subcutaneous fat). Considering the involvement of the *FTO* SNPs in fat accumulation in adults, this physiological strategy seems to indicate that the *FTO* SNPs have experienced evolutionary selection in favor of enhanced thermoregulation at different stages of life.

Indirect selection suggests that temperature might affect other traits thereby indirectly conferring a survival advantage on C-allele carriers, such as susceptibility to certain pathogenic microbes. The variants that confer a stronger immune system or better resistance to certain temperature-associated pathogens may be more beneficial in environments with higher pathogen pressure. Alternatively, C-allele carriers may have a preference for residing in colder regions or be more inclined to actively explore new territories, which could also result in a regional concentration of the derived alleles. Such a pattern may have profound implications for the genetic architecture and subsequent evolution of the species. While the notion of voluntary selection cannot be entirely dismissed at present, it necessitates further empirical substantiation.

The core basis of evolution is the variation of species, namely the diversity of species. Darwinian natural selection theory embodies the principle that survival belongs to those organisms that best fit their environment rather than solely favoring physical strength. In the context of ice-covered earth from millennia ago, the mentioned mutation potentially granted the ability to generate more heat for newborns and infants or thicker insulating subcutaneous fat for adults, thus solidifying its status as a beneficial trait. However, characterized by stable and ample food availability nowadays, its relevance as a “fit” characteristic in the traditional sense may have diminished. Just as the thrifty gene served a pivotal function during years of scarcity, its biological and clinical significance may be misconstrued if evaluated outside of its appropriate historical context—wrongly linking it solely to the adverse implications of diseases, for instance, the positive association of the C-alleles with obesity in adults. Each genetic variation harbors its intrinsic roles; the timing at which its potential to be favored by natural selection and recognized is unpredictable, akin to awaiting a fortuitous turn in the winds of fate.

## Supplementary Material

loae027_suppl_Supplementary_Materials

loae027_suppl_Supplementary_Table_S1

## Data Availability

All data generated or analyzed during this study are included in this article and its supplementary information files. All relevant raw data are available in Supplementary Table S1.
