# Supplementary material for: The FTO variant conferring enhanced UCP1 expression is linked to human migration out of Africa
Source: Life Metab. 2024 Jun 22;3(6):loae027. doi: 10.1093/lifemeta/loae027 (PMC11748519; doi:10.1093/lifemeta/loae027)
Supplement: loae027_suppl_Supplementary_Materials [file loae027_suppl_Supplementary_Materials.docx]

**Supplementary Methods**

**Fetal brown adipose samples**

Human fetal brown adipose samples were obtained from 45 dead fetuses at 20–35 weeks’ gestation after legal medical termination due to developmental malformation or severe hereditary diseases. The gestational ages were estimated by crown–rump length and maternal history. The tissues were collected with informed consent from the parents after their decision to abandon the samples. The protocol was conducted in compliance with the guidelines and approved by the Medical Ethics Committee of Shengjing Hospital of China Medical University (no. 2020PS771K).

**Immunofluorescence analysis**

For immunofluorescence, the paraffin sections of human fetal brown adipose samples were successively stained with UCP1 antibody (ab10983, Abcam; 1:500 dilution) and Perilipin antibody (9349s, CST; 1:200 dilution) using tyramide signal amplification (TSA) strategy with Try-594 and Try-488 as tyramine conversion reagents. Whole-slide scan was produced in an automated acquisition system (TissueFAXS Plus, TissueGnostics).

**RNA extraction and real-time PCR analysis**

Total RNA was extracted from frozen human fetal brown adipose tissue using the Eastep Super Total RNA Extraction kit (ls1040, Promega Biotech). Reverse transcription was performed using the PrimeScript Reverse Transcript Master Mix (RR036A, TaKaRa). Quantitative PCR was performed using a QuantStudio Dx Real-Time PCR Instrument (Applied Biosystems Instrument). The housekeeping gene *36B4* served as the internal control. The primer sets for the human *36B4* gene were forward (5'-CAGATTGGCTACCCAACTGTT-3') and reverse (5'-GGAAGGTGTAATCCGTC TCCAC-3'). The primer sets for the human *UCP1* gene were forward (5'-CAATCACC GCTGTGGTAAAAAC-3') and reverse (5'- GTAGAGGCCGATCCTGAGAGA-3').

**Variant frequency analysis**

Data from a total of 31 distinct ethnic populations were included in this study. Among these, 22 populations were extracted from the 1000 Genomes Project (1KGP) with the effect allele frequency obtained from the online database (https://www.internationalgenome.org). American groups from the 1KGP with recent European admixture were not suitable for our study and were therefore omitted. The remaining 9 populations were obtained from different cohorts with gene frequency data derived from NCBI (https://www.ncbi.nlm.nih.gov/snp/rs1421085" \l "frequency_tab). The determination of the geographical coordinates of the population takes into account the migration characteristics of each population. For populations that are mainly composed of migrants, the coordinates of their long-term residence are selected; for populations whose place of origin and current place of residence are different, the coordinates of their place of origin are uniformly selected. The populations of African ancestry, specifically the ASW (African Ancestry in Southwest USA) and ACB (African Caribbean in Barbados), have been geographically designated using central Africa as a reference point for their ancestral coordinates. For populations of unknown or widespread origin, select the coordinates of their national capital or regional geographic center. The longitude and latitude of each coordinate were determined using Google Satellite Maps. The altitude and annual surface temperature (30-year average) corresponding to each coordinate are obtained from the NASA official website (https://power.larc.nasa.gov/data-access-viewer/). Two temperature-corrected models were employed to simulate the Paleozoic climate to mitigate bias resulting from unmatched SNP selection (which occurred thousands of years ago) and ambient temperatures. All relevant data are available in the **Supplementary Table 1**.

**Statistical analysis**

Data were assessed for normal distribution before performing two-tailed, unpaired Student t-tests. Spearman’s correlation analysis was performed for association analysis. A P value < 0.05 was considered significantly different. The SAS statistical system (v.8.0; SAS Institute, Cary, NC, USA) was used for data analysis. Immunofluorescence intensity were quantified with the ImageJ software v1.51. A topographic map of populations with different genetic variant frequencies was drawn with R (v.3.6.2) and open-source R packages ggplot2, ggmap, RgoogleMaps, sp, maptools, maps, and ggThemeAssist.

**
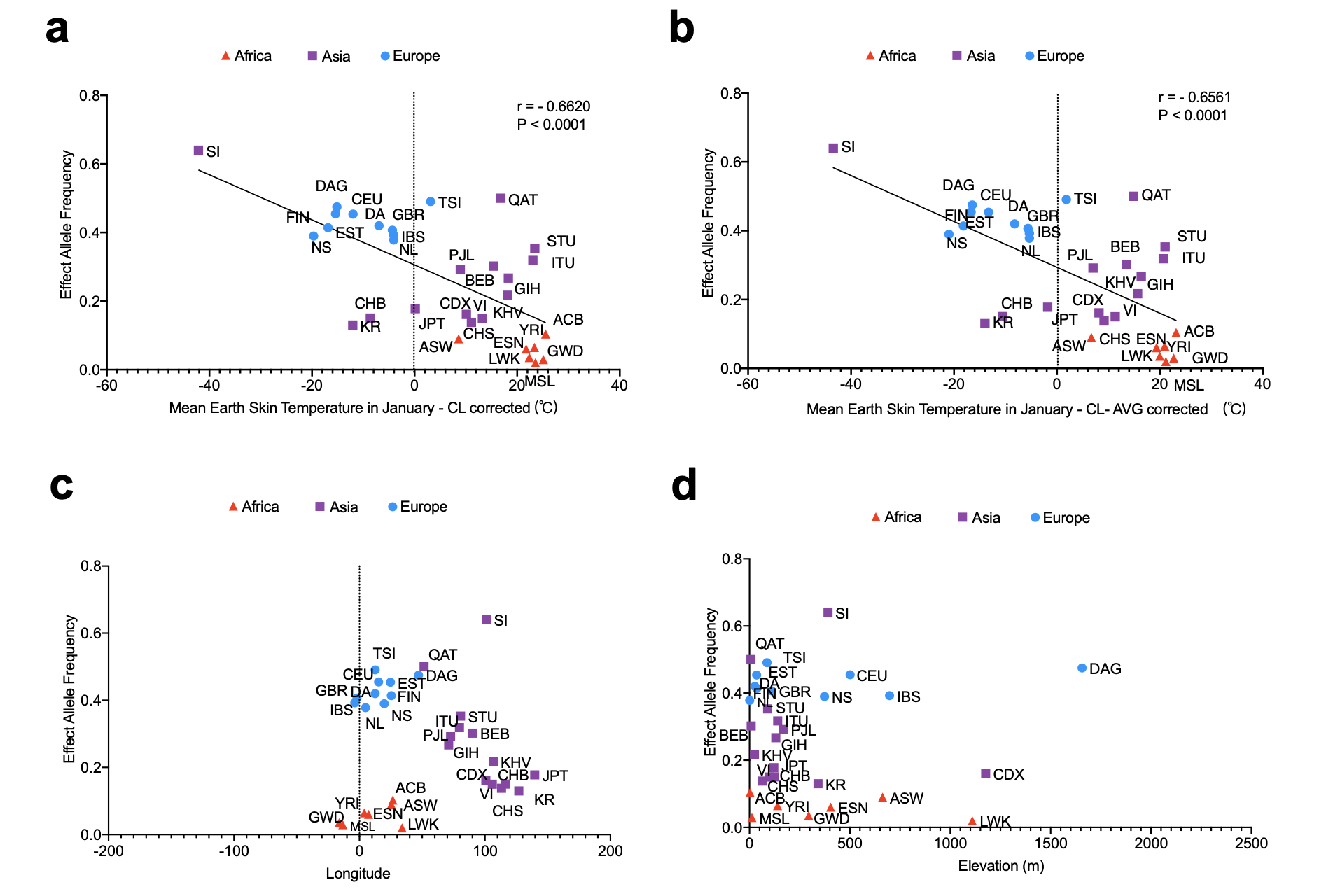
**

**Supplementary figure 1. The association of human *FTO* rs1421085 T>C variation frequency with ambient environmental temperature, longitude, and elevation of population distribution.**

(**a­–b**) The correlation analysis of effect allele frequencies and (Climate: Long-range Investigation, Mapping and Prediction study) CL model-corrected (**a**) or (an average of the CL) CL-AVG model-corrected (**b**) mean earth skin temperature in January. (**c­–d**) The correlation analysis of the frequency of the effect allele (rs1421085_C) and absolute longitude (**c**) or elevation (**d**). Colors and symbols represent populations of different continents.


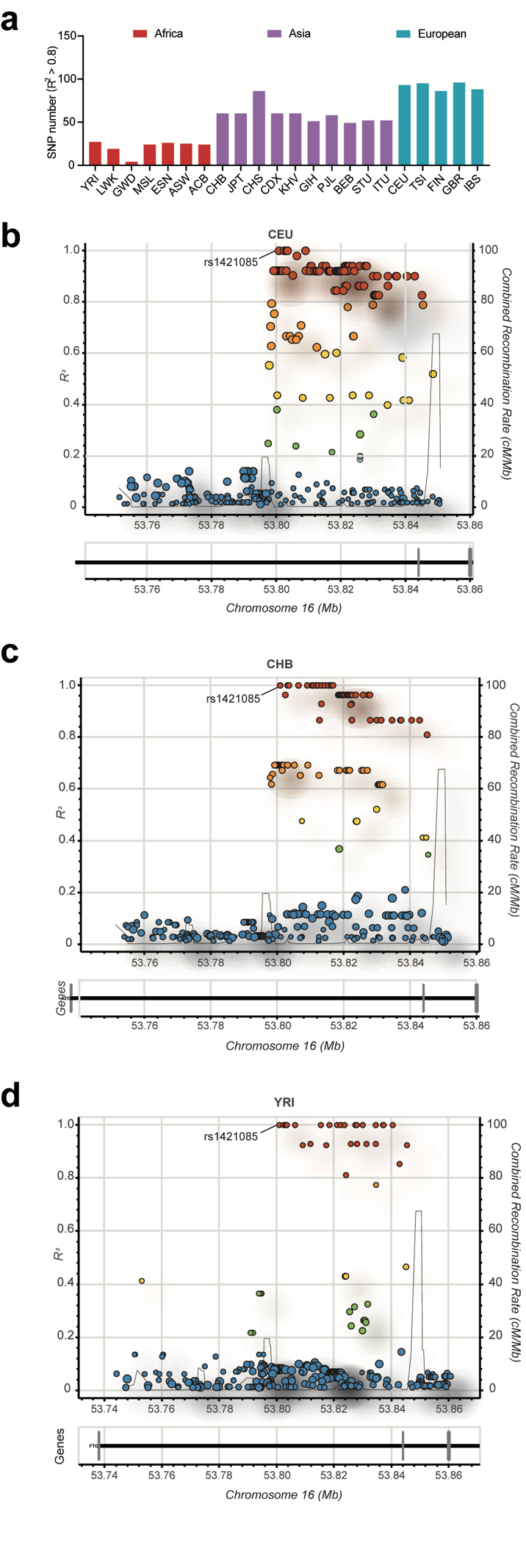


**Supplementary figure 2. Differences in the LD structure of SNPs surrounding rs1421085 among different populations.**

(**a**) The number of SNPs highly linked to rs1421085 differs among people from Africa, Asia and European. (**b­–d**) Regional plots of the *FTO* locus in Utah residents with Northern and Western European ancestry (**b**), Han Chinese in Beijing (**c**) and Yoruba in Ibadan (**d**). SNPs are plotted by position on chromosome 16 and recombination rates reflect the local LD structure. SNPs surrounding rs1421085 are color-coded to reflect their LD with this SNP.
